# Adhesive Performance of Resin Cement to Glass-Ceramic and Polymer-Based Ceramic CAD/CAM Materials after Applying Self-Etching Ceramic Primer or Different Surface Treatments

**DOI:** 10.3390/ma17010002

**Published:** 2023-12-19

**Authors:** Rana Turunç Oğuzman, Soner Şişmanoğlu

**Affiliations:** 1 Department of Prosthodontics, Faculty of Dentistry, Altınbaş University, 34147 Istanbul, Turkey; 2 Department of Restorative Dentistry, Faculty of Dentistry, Istanbul University-Cerrahpaşa, 34098 Istanbul, Turkey; soner.s@hotmail.com

**Keywords:** CAD/CAM materials, surface treatments, self-etching ceramic primer, 10-MDP, bond strength

## Abstract

Ensuring optimum bond strength during cementation is vital for restoration success, with the practicality of the process being crucial in clinical practice. This study analyzed the effect of a single-step self-etching ceramic primer (MEP) and various surface treatments on the microshear bond strength (µSBS) between resin cement and glass-ceramic or polymer-based ceramic CAD/CAM materials. Specimens were fabricated from leucite-based glass-ceramic (LEU), lithium disilicate glass-ceramic (LDC), resin nanoceramic (RNC), and polymer infiltrated ceramic network (PICN) (*n* = 160). They were then classified based on the surface treatments (*n* = 10): control (no treatment); sandblasting with Al_2_O_3_ (AL); etching with hydrofluoric acid (HF); and MEP application. Scanning electron microscopy was used to evaluate the surface topography. µSBS was measured after cementation and thermocycling procedures. Failure modes were examined with a stereomicroscope. Statistical analysis involved two-way analysis of variance and Tukey HSD tests with a significance level of 0.05. µSBS was significantly influenced by both surface treatment and CAD/CAM material type. The most enhanced µSBS values for each material, regarding the surface treatment, were: LEU and LDC, HF; RNC, AL; PICN, AL or HF. MEP significantly increased the µSBS values of CAD/CAM materials except RNC, yet it did not yield the highest µSBS values for any of them.

## 1. Introduction

These systems have become an indispensable part of prosthodontics because they deliver permanent restorations in a single session and thus save time, minimize human error, provide preview by 3D modeling, and produce restorations with excellent fit and precision, superior mechanical properties, and uniform material quality [1,2,3,4].

As a result, to achieve the perfect restoration regarding esthetical, physical, and chemical qualities, the composition of CAD/CAM materials is progressively being improved [5]. The primary categories of contemporary materials include glass ceramics, polycrystalline ceramics, and polymer-based ceramics [1,4]. Within glass ceramics, feldspar ceramic was marketed first. It has a silica-rich phase and excellent aesthetic characteristics [6]. However, its mechanical strength requires improvements, which is achieved by adding crystals like leucite or needle-like particles of lithium disilicate (ranging from 0.5 to 4 µm) to reduce the risk of cracks and bolster the material’s durability and stability [4,5]. Nonetheless, glass ceramics still suffer from brittleness, wear on opposing teeth, the need for post-firing, and challenges in machinability and occlusal adjustments [1,4,5,7]. Hence, new products are being released to address these limitations. One of these new products is polymer-based ceramics, which combine ceramic components with resin monomers and merge the advantages of both. The advantages promoting the quality and survival of restorations can be summarized as enhanced repairability, marginal adaptation, machinability, polishability, lower hardness, elastic modulus, and abrasiveness [3,5,8]. These advantageous polymer-based ceramics can be categorized into two primary classes related to their microstructures: high-temperature polymerized resin-based composites such as resin nanoceramics and polymer-infiltrated ceramic networks (PICNs), also known as hybrid ceramics. Resin nanoceramics comprise composite materials primarily composed of organic phases containing methacrylate monomers and dispersed inorganic filler particles. PICN comprises a porous ceramic network infused with a low-viscosity polymer [1,2,4].

Variations in the chemical composition of CAD/CAM materials influence their bonding performance, determining their success [9]. Adequate bonding increases the fracture resistance of ceramics by allowing equal load distribution through the bonding interface. On the other hand, if the adhesive seal fails, microleakage can occur, which may lead to staining, hypersensitivity, caries, pulpal reactions, and even debonding, shortening the life of the restoration [3,10]. Achieving strong adhesion with restorative materials requires in-depth knowledge of the restorative material, resin cement, and appropriate surface treatment [8,9]. Studies highlight the significance of surface treatment in enhancing the adhesion between the resin cement and CAD/CAM material [7]. The main idea of surface treatment is to create micro-roughness, thereby increasing the surface area. After that, applying a ceramic primer promotes bonding to the hydrophobic luting cement, ensuring micromechanical interlocking and chemical bonding [8,11]. Mechanical or chemical surface treatments are recommended for inert restorative materials to increase that bond strength [5]. 

Sandblasting with aluminum oxide (AL) is commonly used as a practical mechanical treatment, especially for polymer-based restorations. However, its application should be cautious due to potential respiratory irritations. In addition, it can lead to heat production, residual stresses, cracks, microchipping, and volume losses in the ceramic restorative materials that may cause flaws, reducing the bond strength [1,5]. As a chemical surface treatment method, etching with hydrofluoric acid (HF) and conditioning with silane primer is reported to be the gold standard for glass ceramics [10,12,13,14]. HF can induce surface micro-roughness by dissolving the glassy matrix and exposing the crystalline structure, thereby increasing micromechanical retention [3,10,15]. In addition, silane primer improves surface wettability, facilitating a chemical bridge between the glass ceramic’s silica and resin cement’s methacrylate groups, forming a robust bond [10,15]. Different silane coupling agents have been introduced, but the majority contain dilute alcoholic solutions of methacryloxypropyltrimethoxysilane [16]. Recently, silane primers, including 10-methacryloyloxydecyl dihydrogen phosphate (10-MDP), were marketed to interact chemically with ceramics and several other types of restorative materials and thereby maintain the stability of the bond strength [10]. However, HF application along with silane primer is a two-step, technique-sensitive procedure that may risk the bond strength if not applied appropriately. For instance, prolonged use of HF can weaken the ceramic surface and compromise its mechanical properties by forming insoluble silica fluoride salts on the surface, which may weaken the bond strength between the ceramic and the resin cement. In addition, HF is highly dangerous due to its corrosive, reactive, and toxic nature. Direct contact with HF can result in tissue damage, rashes, burns, and even necrosis, so skin and eye protection is mandatory [6,10,13,14]. Moreover, because of this toxic potential, HF ceramic etching is even prohibited in some countries [12].

Manufacturers have sought a safer and more convenient alternative to HF, aiming to eliminate its toxicity, reduce its technical sensitivity, and shorten the duration required for HF [9]. Consequently, Monobond Etch and Prime (MEP; Ivoclar Vivadent AG, Schaan, Liechtenstein) is marketed as a new, advantageous self-etching ceramic primer. In its composition, there is ammonium polyfluoride for gentle etching and trimethoxypropyl methacrylate for silanization, which work simultaneously to enhance the bond strength [13]. It is primarily recommended for glass ceramics, but researchers have also explored its effectiveness on polymer-based ceramics, considering the drawbacks of sandblasting. However, the studies with different experimental setups concluded contradictory results both for glass ceramics and the polymer-based ceramics [1,5,15,17,18,19]. Some researchers suggest MEP demonstrates similar bond strength to HF used with silane, while others indicate HF followed by silane leads to a higher bond strength than that by MEP [5,16,19,20]. In addition, some researchers recommend MEP to treat both glass ceramics and polymer-based ceramics, while others report that MEP promotes adhesion for particular polymer-based ceramics yet may not demonstrate comparable achievement for different polymer-based ceramics, as its performance is material-dependent and further studies are required for different materials [5,17]. Moreover, knowledge of MEP’s performance on bond strength remains scarce compared to other surface treatments. Consequently, this study aimed to assess the influence of MEP and various surface treatments in conjunction with 10-MDP-containing silane primer on the microshear bond strength (μSBS) between the resin cement and various glass-ceramic or polymer-based ceramic CAD/CAM materials. Accordingly, the null hypothesis was set as follows: μSBS is not influenced by the type of surface treatment and CAD/CAM material.

## 2. Materials and Methods

In this in vitro study, four distinct types of CAD/CAM materials were utilized: LEU (IPS Empress CAD; Ivoclar Vivadent AG, Schaan, Liechtenstein), LDC (IPS e.max CAD; Ivoclar Vivadent AG, Schaan, Liechtenstein), RNC (Lava Ultimate; 3M ESPE, St. Paul, MN, USA), and PICN (Vita Enamic; VITA Zahnfabrik H. Rauter, Bad Sackingen, Germany). Comprehensive information on the CAD/CAM materials can be found in Table 1.

### 2.1. Specimen Preparation and Surface Treatments

The CAD/CAM materials were sliced into sections with 2 mm thickness by a precise cutting tool (PRESI, Mecatome T180, Eybens, France). Each section obtained from the CAD/CAM materials was inserted in an auto-polymerizing acrylic resin (Meliodent, Heraeus Kulzer, Hanau, Germany), leaving the surfaces to be tested uncovered. These embedded specimens were meticulously smoothed by employing 600-grit-sized water-cooled silicon carbide papers. Subsequently, the specimens were allocated to the following groups randomly (*n*  =  10):

- Control: No surface treatment was administered.
- AL: Specimen surfaces underwent sandblasting with a micro sandblaster (DENTO-PREP™ Microblaster, Rønvig Dental Products Inc., Daugaard, Denmark) positioned 10 mm away from the surface of the specimen. This was done at a pressure of 2 bars with 50 µm aluminum-oxide (Al_2_O_3_) particles (RondoFlex Preparation Powder; KaVo, Biberach/Riss, Germany). Following treatment, the specimens were soaked in distilled water for 5 min in an ultrasonic bath before being air-dried.
- HF: CAD/CAM material surfaces were subjected to 5% hydrofluoric acid (IPS Ceramic Etching Gel, Ivoclar Vivadent, Schaan, Liechtenstein) for 60 s, except for LDC specimens, which were etched for 20 s in accordance with the manufacturer’s instructions [2,21]. Then, the surfaces were washed off with distilled water and dried with an air spray.
- MEP: Monobond Etch and Prime was kindly rubbed using a microbrush for 20 s. After waiting 40 s, the specimens were washed off and dried with air spray for 10 s, following the manufacturer’s instructions.

### 2.2. Adhesive Cementation and Thermocycling

After the surface treatments, a silane primer (Clearfil Ceramic Primer Plus; Kuraray Noritake, Tokyo, Japan) was applied on the CAD/CAM specimens using an applicator brush. Then, specimens were dried using a mild, oil-free airflow, following the manufacturer’s guidelines. However, the MEP group did not undergo silanization since MEP contains trimethoxypropyl methacrylate, which acts as a silane. Next, a transparent polyvinyl tube was cut meticulously to obtain microtubules with an inner diameter of 1 mm and a height of 0.5 mm, ensuring parallel ends using a gauge. Four resin microtubules were adjusted over each specimen and carefully filled with dual-cure resin cement (Panavia V5; Kuraray Noritake, Tokyo, Japan), resulting in *n* = 10 for each group. They were then polymerized with a blue light-emitting diode (LED) curing unit (Bluephase; Ivoclar Vivadent AG, Schaan, Liechtenstein) in standard mode for 10 s from the top and around the microtubules, following the manufacturers’ instructions. The LED curing unit had a light intensity of 1200 mW/cm^2^, checked with an LED radiometer (L.E.D. Radiometer; Demetron/Kerr, Middleton, WI, USA) prior to polymerization of each group. The polymerized specimens were then stored in distilled water for 24 h at 37 °C in a laboratory oven (FN 055/120; NUVE, Ankara, Turkey) and subjected to thermocycling for 5000 thermal cycles between 5 °C and 55 °C with a 30 s staying period in distilled water for aging.

### 2.3. Microshear Bond Strength Testing

A device utilized for microshear testing (MOD Dental, Esetron Smart Robotechnologies, Ankara, Turkey) having a knife-edged blade was used to measure μSBS by applying a force to the adhesive interface. The crosshead speed was set at 0.5 mm/min, and the failure load was noted in MPa [16]. Subsequently, the modes of failure were observed under a stereomicroscope (SMZ745T; Nikon, Tokyo, Japan) at ×40 magnification. The classification of modes of failure was determined as follows:

- Adhesive (A): When the failure occurs at the interface between different materials.
- Mixed (M): When the failure occurs at the interface between different materials and within the resin cement or ceramic.
- Cohesive in resin cement (CRC): When the failure occurs within the resin cement material.
- Cohesive in CAD/CAM material (CC): When the failure occurs within the CAD/CAM material.

### 2.4. Scanning Electron Microscopy Evaluation

Eight specimens from each CAD/CAM material were processed with surface treatments to execute scanning electron microscope (SEM) analyses, with two samples per treatment group. These specimens were coated with a fine layer of gold using a sputter coater (Polaron SC7620 sputter coater, ThermoVG Scientific, West Sussex, UK). Subsequently, they were scrutinized using SEM (JEOL 5500; JEOL Inc., Peabody, MA, USA) with 10 kV. Analyses were conducted at magnifications of ×1.000 and ×10.000.

### 2.5. Statistical Analysis

The mean values of µSBS data in MPa and their corresponding standard deviations were determined, and the Kolmogorov–Smirnov and Shapiro–Wilk tests were used to find out whether the data distribution was normal. The outcomes of these normality tests indicated that the data followed a normal distribution. Consequently, the influence of different CAD/CAM material types, surface treatments, and the interaction of these variables on µSBS values were investigated using a two-way analysis of variance (ANOVA). Subsequently, the Tukey HSD test was conducted as a post hoc analysis for pairwise comparisons. All statistical analyses were executed utilizing statistical software (SPSS Version 22, IBM, Chicago, IL, USA), with a significance level set at 0.05.

## 3. Results

The type of CAD/CAM material and the surface treatment had a statistically significant influence on the μSBS values between the CAD/CAM materials and resin cement, according to the results of the two-way ANOVA displayed in Table 2. In addition, between these two variables, a statistically significant interaction was found (*p* < 0.001).

The mean μSBS values with standard deviations following the surface treatments are displayed in Table 3. Control groups of all CAD/CAM materials, except MEP-treated RNC, demonstrated significantly lower μSBS than the surface-treated groups. In the pairwise analysis of the surface-treated LEU groups, no significant difference was found. In addition, there was no significant difference between HF and MEP for LDC specimens, but AL resulted in significantly lower values than HF. Furthermore, regarding RNC specimens, AL enhanced the μSBS significantly more than HF, and HF yielded significantly higher μSBS than MEP. In addition, PICN specimens treated either with AL or HF demonstrated significantly greater μSBS values, followed by MEP. Regarding the surface treatment, AL treatment resulted in the greatest μSBS for RNC and PICN in comparison with the other CAD/CAM materials. However, HF and MEP resulted in significantly the lowest μSBS values for RNC, and there was no other significant difference in terms of surface treatment among the other CAD/CAM materials.

When the distribution of failure modes was evaluated, it was seen that control groups for all CAD/CAM materials showed adhesive failure without any exception (Table 4). The highest percentage of mixed and cohesive failures observed for LEU was in HF-treated specimens, followed by MEP and AL, parallel with the μSBS values displayed in Table 3. Similarly, for LDC specimens, the frequency of mixed and cohesive failures was higher in HF-treated specimens, followed by MEP. However, for RNC specimens, mixed and cohesive failures were higher in AL, followed by HF and MEP. For PICN specimens, HF and AL treatments showed higher rates of mixed and cohesive failures. However, when compared to AL, HF demonstrated more cohesive failures, half of which were cohesive failures within the ceramic. The other groups displaying failure within ceramic were RNC treated with AL and LEU treated with HF.

**Figure 1:**
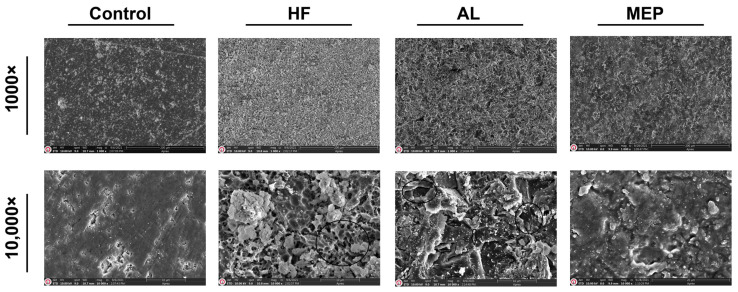
SEM images of LEU after different surface treatments at 1000× and 10,000× magnifications. The marked area in the HF-treated sample’s image with 10,000× magnification resembles the honeycomb pattern. The areas pointed in circles in the AL-treated sample’ image with 10,000× magnification are examples of craters, and the arrows show the depressed and elevated layers.

**Figure 2:**
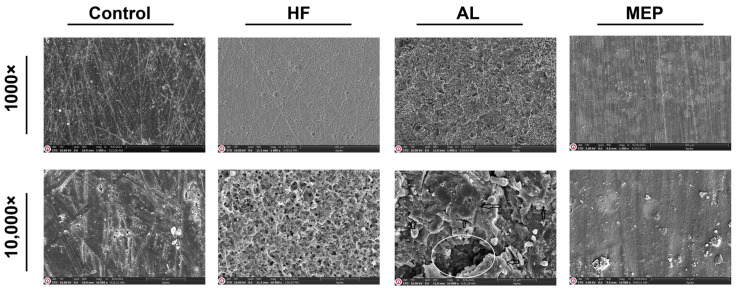
SEM images of LDC after different surface treatments at 1000× and 10,000× magnifications. The area in the circle in the AL-treated sample image with 10,000× magnification is an example of a crater, and the arrows show depression and elevation areas.

**Figure 3:**
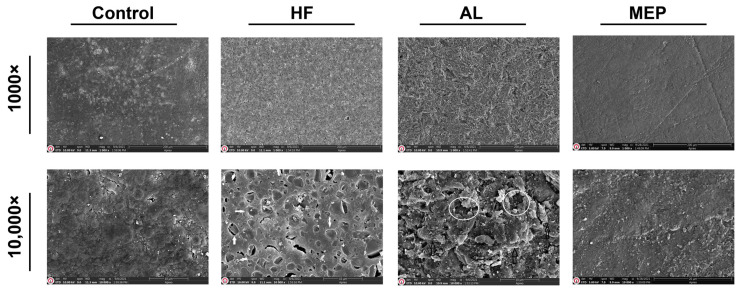
SEM images of RNC after different surface treatments at 1000× and 10,000× magnifications. The white arrows in the HF-treated sample image with 10,000× magnification indicate the shallow pits. In the AL-treated sample image with 10,000× magnification, the areas in circles show craters and the arrows indicate depression and elevation areas with undercuts and grooves.

**Figure 4:**
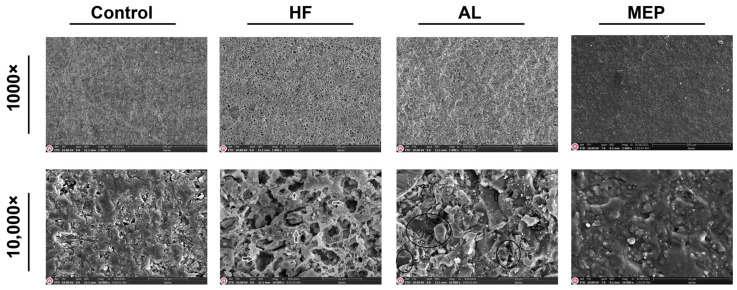
SEM images of PICN after different surface treatments at 1000× and 10,000× magnifications. The arrows in the HF-treated sample image with 10,000× magnification indicate the deep pores. The circles in the AL-treated sample image with 10,000× magnification show the craters, and the arrows indicate the sharp-edged geometry with crevices.

SEM micrographs in Figure 1, Figure 2, Figure 3 and Figure 4 depict the surface conditions of CAD/CAM materials following various surface treatments. Comparatively, the control groups for each material displayed smoother surfaces when contrasted with the AL and HF treatment groups. In contrast, the surface topography of the control group specimens seemed similar to that of the MEP treatment groups. AL treatment induced pronounced craters, resulting in depressed and elevated layers across all CAD/CAM materials. Conversely, HF treatment generated a relatively microporous surface reminiscent of a honeycomb pattern, especially evident on glass ceramics. Notably, HF treatment on LEU, LDC, and PICN revealed dissolution of the glassy phase, resulting in deep pores capable of accommodating primer and resin penetration. In contrast, RNC specimens subjected to HF exhibited shallower pits rather than pores. However, when treated with AL, RNC specimens exhibited increased irregularities characterized by undercuts, grooves, and craters on the material surfaces. In addition, AL-treated PICN specimens displayed bound porosities with crevices, displaying a more sharply edged microgeometry.

## 4. Discussion

Cementation is a crucial process for the restoration’s longevity, requiring severe caution. That is because if the adhesion is not adequately achieved and sustained, microleakage can occur, which may result in hypersensitivity, caries, pulpal reactions, a decrease in the fracture resistance of ceramics, and even debonding [3,10]. Thereby, researchers try to set the best cementation protocol in terms of practicality and bond strength for restorative materials. Regarding this, the current study aimed to investigate the influence of single-step MEP and various surface treatments in conjunction with MDP-containing silane primer on the μSBS between the resin cement and various glass-ceramic and polymer-based ceramic CAD/CAM materials. In terms of bond strength, the ANOVA test revealed that the μSBS was influenced by both the type of surface treatment and CAD/CAM material, so the null hypothesis was rejected. 

Many researchers reported that the type of surface treatment, restorative material, and surface conditioning materials are essential to determine the success of bond strength between the resin cement and CAD/CAM material. As one of the surface treatments, AL improves micromechanical interlocking between the CAD/CAM material and the resin cement by modifying the surface by promoting its roughness, area, energy, and wettability [2,22]. The SEM images (Figure 1, Figure 2, Figure 3 and Figure 4) also demonstrated that AL changes the surface topography significantly by resulting in microcavities. Supportively, the μSBS results also showed that AL enhanced all the restorative materials’ bond strength significantly, and it even yielded the highest μSBS values for RNC compared with the other surface treatments. Additionally, it provided significantly the most enhanced μSBS values along with HF for PICN material. Previous studies reported that AL significantly increases the roughness and surface energy in polymer-based ceramics and causes a moderate increase in glass ceramics, thus increasing the bond strength [7,23,24]. The possible explanation for increased bond strength related to polymer-based ceramics is that AL exposes the inorganic fillers in the polymer matrix, facilitating the development of siloxane bonds between these fillers and the silanol present in the silane primer [21]. Consequently, AL-treated polymer-based ceramics exhibited a higher rate of mixed and cohesive failures, meaning the adhesive force surpasses the cohesive force within the restorative material or cement, signifying reliable bond strength and successful cementation [6,25,26]. Along with these advantages, AL also has some drawbacks, including the potential to cause microcracks in ceramics and its technical sensitivity due to various application parameters like particle type, size, pressure, duration, and angle [7,22]. Moreover, AL did not significantly increase the µSBS between the resin cement and the glass ceramics, particularly LDC, compared to HF.

HF dissolves the glassy phase in restorative materials, increasing the surface roughness and wettability while reducing the contact angle. This contributes to microretention by releasing hydroxyl groups that can bind with monomers [27,28]. Therefore, as found in this study, it provides a more effective binding in glass ceramics or PICN materials with a more glassy phase. Deep microchannels with honeycomb-like patterns in the SEM images (Figure 1, Figure 2 and Figure 4) and the increase in cohesive failures (Table 4) also support this result. In addition, previous research also revealed that HF followed by silane application increases the hydrophilicity of the resin cement and enhances the physicochemical reactions between it and the ceramic, and this application is regarded as the gold standard for glass ceramics [19,25]. On the other hand, HF did not increase the bond strength between RNC and resin cement as significantly as AL. The differences in μSBS among the polymer-based ceramics are attributed to the differences in the materials’ compositions, type, size, and ratio of the inorganic fillers (RNC has 80% inorganic fillers while PICN has 86%), crystalline phase, and manufacturing method, which cause varying degrees of wettability and hydrophilicity [4,7,11,19,24]. In addition, as can be seen in the SEM images, HF caused a wider but shallower porosity (Figure 3) on RNC compared to the other CAD/CAM materials. Since these pores were not as deep as those in the others, silane and cement infiltration could not be achieved sufficiently, affecting μSBS adversely [15]. Some previous studies and manufacturers have also recommended AL for RNC and HF for PICN, in line with the present study [7,11]. The reason for not recommending HF for RNC was that HF weakens adhesion as it completely dissolves the inorganic phase in the resin matrix [7,11]. However, HF treatment is still tested on RNC in the present study since there are also previous studies recommending HF treatment for RNC as it achieves a bond strength greater than or equal to AL treatment [2,29]. These studies had such results probably due to the lack of aging and differences in the test design [2,11,29]. Similar to the current study, a previous study reported that the RNC is not sufficiently affected by HF because the zirconia crystalline phase in the RNC is acid-resistant, so the bond between the resin cement and RNC can be increased with 10-MDP-containing silane [7]. Silane application increases the wettability and hydrophilicity of the resin cement, enhancing the physicochemical reactions between it and the ceramic, and is considered the gold standard following HF for glass ceramics [8,19,25,30]. Silane surrounds silica with methacrylate double carbon bonds that can copolymerize with the resin matrix, enhancing the connection between inorganic filler and resin matrix [2]. It acts as a bridge by creating a siloxane network on the ceramic surface [25,31]. However, conventional silane primers are not sufficiently effective on metal oxides such as zirconia, so 10-MDP-containing silane with a high affinity for metal oxides was used in this study [32]. The 10-MDP molecule comprises a phosphoric-acid group on one end, a vinyl group on the other, and a spacer ester chain of ten carbons between them. The vinyl group promotes polymerization and forms chemical bonds with unsaturated carbon links in the substrate’s resin matrix. The phosphate group in 10-MDP plays a vital role in enhancing bonding with metal oxides like zirconia [33]. Although HF combined with 10-MDP-containing silane effectively increases the bond strength, mainly when used with silane, new alternatives are needed because HF is toxic, requires safety precautions, is multi-stage, requires technical precision, and is time consuming [7,34]. 

Given the disadvantages of HF, manufacturers focused on simpler and safer alternatives and introduced the single-stage MEP, which simultaneously acidifies and silanates, thus minimizing the margin for error [19]. According to the results of this study, MEP leads to higher bond strength than AL and lower bond strength than HF in glass ceramics, but no significant difference was found between MEP and HF. Previous studies have obtained similar results, but some reported that HF gave higher results, although not significantly, while others reported that MEP increased the bond strength more [6,10,13,15,16,19,35]. The variations arise from factors such as whether aging was performed, the method and duration of aging if applied, the use of ultrasonic bath for post-surface treatment cleaning, alcohol utilization in the bath, differences in silanes and resin cement, variations in HF concentration and application duration, the utilization of different bonding strength tests, or the testing of diverse load applications [3]. Despite the similarity in surface topography between the control group and MEP-treated groups observed under SEM, the increase in bond strength is attributed to the direct penetration of silane into the porosities created by the acidic component in MEP and the retention of silane in a thin layer on the surface after rinsing with air-water spray [15,21]. However, MEP did not show a significant difference in bond strength compared to the control group for RNC. On the other hand, it resulted in a significant increase for PICN compared to the control, although it did not perform as well as AL or HF combined with silane. Parallelly, another study indicated that the HF + silane application remained the gold standard for PICN, providing significantly higher bonding strength than MEP [36]. MEP’s lower bond strength could be attributed to its weaker acid (pH = 3.8) compared to HF (pH = 2). Nevertheless, when analyzing failure patterns, an increase in cohesive failures within the resin cement is observed with MEP application. This suggests that bonding strength is not the sole factor determining the failure type, and the mechanical strength of the resin cement also plays a significant role in this regard [37].

Previous studies investigating the effect of MEP on bond strength have primarily focused on glass ceramics, with limited research on its effect on polymer-based ceramics. Moreover, comparative surface treatments were either not combined with silane or combined with mostly 10-MDP-free silane. Therefore, in this study, the impact of silane containing 10-MDP was observed in both glass ceramics and polymer-based ceramics, particularly in RNC containing zirconia. When the findings were evaluated, it was observed that MEP did not yield the highest bonding strength results for any CAD/CAM material. Additionally, previous studies have reported a clinically acceptable bonding strength range of 15–25 MPa. Therefore, the safe surface treatment combinations with 10-MDP-containing silane for the materials used in this study are as follows: LEU, HF; LDC, HF; RNC, AL; PICN, AL, or HF. Nevertheless, considering the disadvantages of these surface treatments, MEP can still be preferred for retentive crown preparation with an ideal taper and surface area since bond strength does not solely depend on the cement [38,39].

This study has several limitations. Firstly, it is an in vitro study, lacking factors like saliva, biofilm activity, occlusal forces, pH, and temperature changes [15]. However, 5000× thermal cycles after cementation were applied to compensate for thermal stresses partially. This procedure accelerates hydrolytic degradation due to contraction and expansion stresses caused by varying thermal expansion coefficients among materials. This phenomenon is vital for predicting the adhesive performance of restorative interfaces. [19]. Secondly, the study focused solely on the impact of surface treatments on ceramic materials, neglecting considerations for the tooth/cement interface in clinical settings. 

In future studies, different resin cements, restorative materials, surface treatment applications, and aging methods, including mechanical ones and more extended aging periods, should be investigated. Furthermore, the possible influence of MEP and other surface treatments on the optical properties could also be studied. Moreover, the findings of laboratory studies should be validated through clinical trials. 

## 5. Conclusions

Within the confines of this study’s limitations, the following conclusions were drawn:

- The effectiveness of the surface treatment method on bond strength is mainly material dependent.
- Mechanical roughening by sandblasting is more effective in enhancing bond strength for polymer-based ceramics than glass ceramics.
- Chemical etching with HF resulted in the highest μSBS for glass ceramics and PICN material in terms of enhancing bond strength.
- MEP is more effective for glass ceramics such as leucite or lithium disilicate-reinforced glass ceramics, but it does not enhance bond strength as much as HF.

## Figures and Tables

**Table 1:** Materials used in the study.

Material	Lot. No.	Type	Composition	Manufacturer
LEU	Y45442	Leucite-based glass-ceramic	SiO_2_ (60–65 %wt), Al_2_O_3_ (16–20 %wt), K_2_O (10–14 %wt), Na_2_O (3.5–6.5 %wt), other oxides (0.5–7 %wt), pigments (0.2–1 %wt).	IPS Empress CAD; Ivoclar Vivadent AG, Schaan, Liechtenstein
LDC	Z00921	Lithium disilicate glass-ceramic	SiO_2_ (57–80%), Li_2_O (11–19%), K_2_O (0–13 %wt), P_2_O_5_ (0–11%), ZrO_2_ (0–8%), ZnO (0–8%), Al_2_O_3_ (0–5%) MgO (0–5%), coloring oxides (0–8% by weight).	IPS e.max CAD; Ivoclar Vivadent AG, Schaan, Liechtenstein
RNC	N619802	Resin nanoceramic	Bis-GMA, UDMA, Bis-EMA, TEGDMA. Filler: ZrO_2_ (4–11 nm) and SiO_2_ (20 nm), aggregated zirconia/silica cluster filler, 80% by weight.	Lava Ultimate; 3M ESPE, St. Paul, MN, USA
PICN	51540	Polymer-infiltrated ceramic network	UDMA, TEGDMA. Filler: Feldspar ceramic enriched with aluminum oxide, 86% by weight.	Vita Enamic; VITA Zahnfabrik H. Rauter, Bad Sackingen, Germany
MEP	Z01RL2	Self-etching glass-ceramic primer	Butanol, tetrabutylammonium dihydrogen trifluoride, methacrylated phosphoric acid ester, trimethoxypropyl methacrylate monomer, ethanol, colorant, water.	Monobond Etch and Prime; Ivoclar Vivadent AG, Schaan, Liechtenstein
Clearfil ceramic primer plus	4F0078	Universal prosthetic primer	3-MPS, ethanol, 10-MDP.	Kuraray Noritake, Tokyo, Japan
Panavia V5	4R0197	Dual-cure resin cement	Bis-GMA, TEGDMA, hydrophobic aromatic dimethacrylate, hydrophilic aliphaticdimethacrylate, initiators, accelerators, silanated barium glass filler, silanated fluoroaluminosilicate glass filler, colloidal silica, silanated aluminum oxide filler, dl-camphorquinone, pigments.	Kuraray Noritake, Tokyo, Japan

Abbreviations: 2-hydroxyethyl methacrylate (HEMA), 3-methacryloyloxypropyl trimethoxysilane (3-MPS), 10-methacryloyloxydecyl dihydrogen phosphate (10-MDP), bisphenol A polyethylene glycol diether dimethacrylate (Bis-EMA), bisphenol A diglycidylmethacrylate (Bis-GMA), triethylene glycol dimethacrylate (TEGDMA), urethane dimethacrylate (UDMA).

**Table 2:** Influence of CAD/CAM material type and surface treatment on µSBS results according to the two-way ANOVA.

Source	Type III Sum of Squares	df	Mean Square	F	Sig.
Corrected model	2812.831 ^a^	15	187.522	45.664	0.000
Intercept	22,313.814	1	22,313.814	5433.696	0.000
CAD/CAM material	76.393	3	25.464	6.201	0.001
Treatment	2304.170	3	768.057	187.032	0.000
CAD/CAM material × treatment	432.269	9	48.030	11.696	0.000
Error	591.345	144	4.107		
Total	25,717.990	160			
Corrected total	3404.176	159			

^a^ R squared = 0.826 (adjusted R squared = 0.808).

**Table 3:** Means and standard deviations of µSBS data in MPa and post hoc analysis for pairwise comparison.

	CAD/CAM Materials			
	LEU	LDC	RNC	PICN
**Control**	4.2 ± (1.2) ^A, b^	5.8 ± (1.5) ^A, c^	6.4 ± (1.3) ^A, c^	5.7 ± (2.0) ^A, c^
**AL**	12.8 ± (1.3) ^B, a^	12.8 ± (1.6) ^B, b^	16.7 ± (3.3) ^A, a^	16.1 ± (2.5) ^A, a^
**HF**	15.6 ± (1.2) ^A, a^	16.2 ± (2.2) ^A, a^	12.0 ± (1.4) ^B, b^	16.2 ± (1.9) ^A, a^
**MEP**	13.4 ± (1.4) ^A, a^	14.9 ± (1.2) ^A, ab^	8.3 ± (2.9) ^B, c^	12.2 ± (2.2) ^A, b^

Different superscript uppercase letters in the same row imply significant differences among CAD/CAM materials and different superscript lowercase letters in the same column imply significant differences among surface treatments according to the pairwise analysis (*p* < 0.05). Abbreviations: AL, aluminum-oxide sandblasting; HF, hydrofluoric acid etching; LEU, IPS Empress CAD; LDC, IPS e.max CAD; MEP, Monobond Etch and Prime application; PICN, Vita Enamic; RNC, Lava Ultimate.

**Table 4:** Failure mode distribution.

		Failure Mode			
CAD/CAM Material	Surface Treatment	A	M	CRC	CC
LEU	Control	10	0	0	0
	AL	8	1	1	0
	HF	3	3	3	1
	MEP	4	3	3	0
LDC	Control	10	0	0	0
	AL	9	1	0	0
	HF	3	5	2	0
	MEP	5	4	1	0
RNC	Control	10	0	0	0
	AL	4	2	3	1
	HF	6	1	3	0
	MEP	6	2	2	0
PICN	Control	10	0	0	0
	AL	4	4	2	0
	HF	4	2	2	2
	MEP	5	2	3	0

Abbreviations: AL, aluminum-oxide sandblasting; A, adhesive; CC, cohesive in ceramic; CRC, cohesive in resin cement; HF, hydrofluoric acid etching; LEU, IPS Empress CAD; LDC, IPS e.max CAD; MEP, Monobond Etch and Prime application; M, mixed; PICN, Vita Enamic; RNC, Lava Ultimate.

## Data Availability

The data presented in this study are available on request from the corresponding author.
